# Multisensory hallucinations and other unusual sensory experiences in the context of migraine: a systematic review

**DOI:** 10.1007/s00415-023-12144-9

**Published:** 2024-01-18

**Authors:** Yixuan Li, Caitlin O. B. Yolland, Susan L. Rossell, Iris E. C. Sommer, Wei Lin Toh

**Affiliations:** 1 Centre for Mental Health & Brain Sciences, Swinburne University of Technology, P.O. Box 218, Hawthorn, VIC 3122 Australia; 2 Academic Services, Boundless Learning, Melbourne, VIC Australia; 3 Department of Psychiatry, St Vincent’s Hospital, Melbourne, VIC Australia; 4 Department of Psychology, Alfred Hospital, Melbourne, VIC Australia; 5 Department of Biomedical Sciences of Cells and Systems, University Medical Center Groningen, Groningen, The Netherlands; 6 Graduate School of Medical Sciences (Research School of Behavioural and Cognitive Neurosciences), University of Groningen, Groningen, The Netherlands

**Keywords:** Migraine, Aura symptoms, Non-visual, Hallucinations, Unusual sensory experiences

## Abstract

**Objective and background:**

Visual auras in migraine have been extensively studied, but less is known about multisensory hallucinations or other unusual sensory experiences, including whether these should be diagnostically considered as part of aura symptoms. The current study aimed to conduct a systematic review and synthesis to bring together existing empirical evidence on these non-visual perceptual experiences, focusing on their phenomenological descriptions and clinical correlates.

**Methods:**

Forty-eight relevant studies were included based on a systematic search across PsycINFO APA and Web of Science, for peer-reviewed publications in the English language, from 1980 to the present. These comprised a mix of case reports/series (*n* = 19) and group design studies (*n* = 29).

**Results:**

Reports of complex multisensory hallucinations, beyond typical established aura symptoms, were numerous and varied in nature. Yet there were limited data on how this related to patient distress and functional interference. Other sensory distortions or hypersensitivities across non-visual domains were also evident, and generally more common in those with established aura symptoms**.**

**Conclusion:**

Our findings provide preliminary evidence that multisensory hallucinations and other unusual perceptual experiences in migraine are likely more common than previously believed. Further investigations are needed to appropriately account for these symptoms within current nosological systems. Increased clinician–patient awareness is important for managing distress (where necessary), and potentially for offering a holistic therapeutic approach to migraine management.

**Supplementary Information:**

The online version contains supplementary material available at 10.1007/s00415-023-12144-9.

## Introduction

Migraine is a common disorder, often characterised by episodes of pulsating unilateral headaches, as defined in the International Classification of Headache Disorders, third edition (ICD-3; Headache Classification Committee of the International Headache Society (IHS) [1]). The Global Burden of Disease study ranked migraine as the sixth-most prevalent and among the top 10 most disabling diseases, affecting approximately 14.4% of individuals worldwide [2]. Migraine can be diagnosed as with or without aura, which refers to completely reversible central nervous system disturbances [1]. Six types of aura symptoms are described, namely, involving visual, sensory, speech and/or language, motor, brainstem or retinal disturbances. Most common are visual aura symptoms, examples of which can include zigzag figures at a central fixation point, scotoma involving blind spots or scintillating scotoma characterised by flickering lights or colours.

While complex visual phenomena are currently also included as aura symptoms, hallucinations or other unusual sensory experiences in non-visual modalities have not been accounted for. “Sensory aura” implies that other senses may be involved, but only somatic events involving numbness or tinging, or pins-and-needles have been well described; auditory, olfactory, gustatory, or complex tactile experiences are seemingly absent within existing diagnostic criteria. Yet literature has suggested that these latter non-visual experiences are in fact, not uncommon in migraine [3–5]. As the ICHD-3 nosology has focused on visual and somatic aura symptoms like those outlined, most empirical studies have been in these areas [6]. In contrast, research regarding the phenomenology of complex hallucinations or other unusual perceptual experiences involving non-visual modalities has been relatively limited.

Understanding these experiences has clear clinical implications for awareness, diagnosis and management for both individuals with migraine and their clinicians. Nosologically, it is important that the disease classification captures typical migraine symptoms as well as less common aura experiences, to ensure accurate and inclusive diagnosis. Having a comprehensive understanding of the range of symptoms experienced in migraine is also important for investigations of complex neurobiological mechanisms contributing to onset. Finally, precision medicine has been highlighted as a critical future step in migraine diagnosis and intervention [7], for which a complete picture of symptoms at hand is needed.

The present study aimed to bridge these gaps in existing literature, by systematically reviewing and synthesising past research regarding multisensory hallucinations and other unusual sensory experiences in migraine. The following primary research questions were posed: (i) what was the phenomenology of complex multisensory hallucinations as potential migraine aura symptoms? And (ii) what other multisensory unusual perceptual experiences, distinct from complex hallucinations, may be identified? A third aim was to explore whether there were notable patterns or trends in migraine symptoms, including frequency, as associated with manifestation of these hallucinatory or other unusual sensory events, within our included samples.

## Methods

The review adhered to guidelines described in the Preferred Reporting Items for Systematic Reviews and Meta-Analyses (PRISMA) statement [8]. The study protocol was pre-registered on the International Prospective Register Reviews (PROSPERO; CRD42022298603).

### Search strategy and selection criteria

A systematic search was conducted across PsycINFO and Web of Science, for peer-reviewed publications in English, from 1980 to the present, with a cut-off date of 1st February 2023. Search terms were focused on two primary keywords/phrases: “hallucinations” or “unusual sensory experiences”, based on non-visual domains in the context of migraine. Optimised permutations of search terms were used (see Table A in Supplementary materials for search syntax by database).

Articles were screened based on the following inclusion criteria: (i) main outcome variable(s) involving hallucinations or unusual sensory experiences outside of established aura symptoms or known prodromal or postdromal indicators (e.g. photophobia or phonophobia). Exclusion criteria were as follows: (i) no diagnosis of migraine; (ii) participants aged under 12 years old; (iii) significant neurological comorbidity causing migraine (e.g. tumours or brain injury) and (iv) replicated datasets (for which the single most appropriate paper was included to avoid duplication).

### Study selection and data extraction

**Fig. 1:**
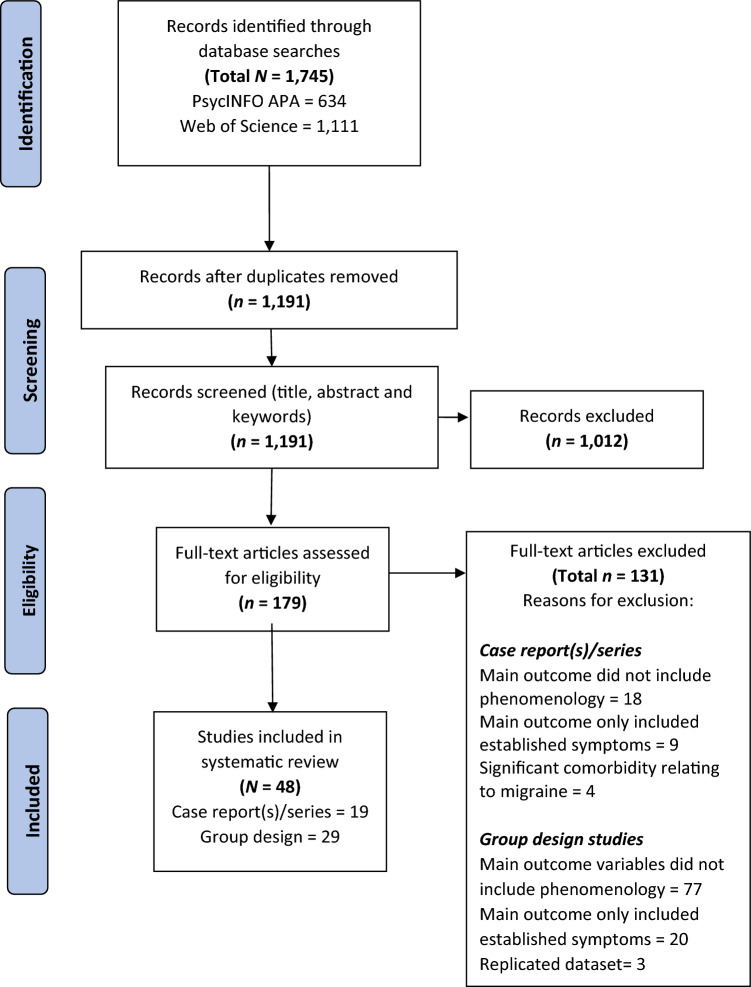
PRISMA flow diagram

After removing duplicates, YL screened the title, abstract and keywords of retrieved publications, and excluded irrelevant studies. A review of full-text articles was then conducted independently by CY and YL using the inclusion/exclusion criteria outlined. Discussions with senior author WLT were conducted to resolve any discrepancies in study selection between YL and CY. The search strategy yielded a total of 1745 records, from which *n* = 48 (case studies = 19, group design studies = 29) met inclusion criteria in the final stage. Figure 1 shows the PRISMA flow diagram.

Data extraction was jointly performed by CY and YL and comprised study identifiers (author(s), year of publication), participant characteristics (e.g. age, sex, subgroup sizes), phenomenological descriptions of established aura symptoms (including visual and sensory aura), complex hallucinatory experiences, prodromal or postdromal symptoms and other unusual sensory experiences (otherwise unaccounted for).

### Risk of bias assessment

A risk of bias assessment was conducted by YL using the JBI Critical Appraisals Checklist for Case Studies (JBI-CAC; Munn [9]) and Newcastle–Ottawa Quality Assessment Scale (NOQAS; [10] for group design studies, adapted for our purpose (see Tables C and D in Supplementary materials). One item was added to the JBI-CAC to assess descriptive information, as this review was focused primarily on the phenomenology of hallucinations or unusual sensory experiences in the context of migraine; items relevant to interventions or treatment were removed. Two items were added to the NOQAS to assess the validity of hallucination and migraine symptom assessments. Within each of the seven domains, 0–2 points were awarded, based on how well each response satisfied the criterion under assessment (nb. only 0–1 points could be awarded for the item “Representativeness of the cases” for NOQAS). The unweighted summed scores provide an indication of the overall quality and bias of each study. Categorical ratings were devised as follows: *Excellent* 10–12/12, good 7–9/12, *fair* 4–6/12 and *poor* ≤ 3/12 for the JBI-CAC and NOQAS*.*

## Results

Results from case and group design studies were extracted and summarised separately, owing to disparate aims and variables employed across these distinct types of empirical research.

### Multisensory hallucinatory and other unusual sensory experiences: case studies

Descriptive phenomenology of multisensory and other unusual sensory experiences in migraine extracted from case studies (*n* = 19) is shown in Table 1. To contextualise these findings, we opted to present participant characteristics, comprising age, sex, neurological and psychiatric comorbidities as well as associated migraine symptoms and their timelines, where available. A wide participant age range was observed (12–72 years old), with slightly more males (56%) than females overall. Neurological and psychiatric comorbidities varied, ranging from focal lesions to epilepsy for the former and bipolar or depressive disorders for the latter. Yet significant neurological or psychiatric histories (including psychotic illness) were excluded for about half of the included studies (*n* = 10).

**Table 1:** Multisensory hallucinatory and other unusual sensory experiences in migraine (*n* = 19 case studies)

References	Participant age in years and sex (and included *n* for case series)	Neurological and/or psychiatric comorbidity	Established aura symptoms^a^ (including visual; complex visual hallucinations presented in next column)	Description of complex hallucinatory experiences (visual, auditory, olfactory, somatic-tactile, gustatory and other lesser-known modalities)	Other unusual sensory experiences		Associated migraine symptoms	Timeline relative to headache (if present)
					Prodromal or postdromal (sensory) symptoms^b^	Others		
Alstadhaug and Benjaminsen [22]	40f	Bipolar disorder, Type II	Brief central grey scotoma; flickering or sparkling at rim of upper lateral visual field	Intense taste of lemons accompanied by salivary flow	None	Synaesthesia lasting 20–30 min	6-year migraine history, reduced from 2 to 1 weekly episode with *valproate*	Before (1 h pain-free interval)
Barros et al. [23]	48m, 38f (2/7)	Right central facial palsy (38f)	Scintillating scotoma (4/7); zigzag lines (1/7)	Illusion of time travel, being sucked into a spiralling tunnel leading to a luminous, serene paradise and sent back after a few minutes; also sense of being followed	Photophobia; phonophobia (5/7)	None	Migraine onset at age 9–15, variable headache duration (30 min–72 h), moderate pain intensity with throbbing or tightening quality	Unknown
Bhatia et al. [24]	30m	No epilepsy or TBI history	Bilateral photopsia lasting 30 min; scintillating scotoma; numbness in right arm ascending to face in perioral distribution; left hemiparesis	Sensation of objects crawling over four limbs; feeling objects revolving and shimmering as if on a giant wheel	Photophobia	None	Migraine onset at age 14, > 50 lifetime episodes	Before (30 min pain-free interval); during
Burstein et al. [25]	42m	Unknown	Scintillating scotoma; blurred/distorted vision; numbness and tingling in arm and face, settling in lips and part of tongue contralateral to headache; tinnitus	Off-putting odours; bitter and unpleasant tastes	Photophobia; phonophobia	Osmophobia	Migraine onset at age 14, 1–2 episodes per month, lasting 2–6 h (12–24 h if untreated), unilateral headaches on either side, > 7/10 pain intensity, with throbbing quality	Before (30–60 min pain-free interval); during (gustatory)
Chen et al. [26]	51f	No TBI history	None	None	Photophobia	Hypoesthesia in left hand and upper limb lasting 20–30 min (resolved with menopause)	Migraine onset at age 46, monthly episodes co-occurring with menstruation, lasting 6 h, with holocranial, throbbing, outward-expanding headaches	Before (no pain-free interval)
Donat and Donat [27]	35f	Unknown	None	Smell of oranges or coffee, lasting several minutes	Photophobia	None	Migraine onset at age 11, lasting hours, unilateral pain on either side, with nausea	Before
Fisher [28]	44f	None	Fortification spectra; photopsia; scintillating scotoma; left-sided numbness spreading from head to feet causing aphasia; tingling on face and tongue lasting 2–5 min	Described built-in personal television screen showing different scenes; buzzing sounds; odour of dead cat or rotting animals at night, “resembling an awful stench-like death” or “beautiful” ammonia smells	None	Temporary loss of vision lasting days; “electric shocks” affecting arm, shoulder and head	Migraine onset at age 7, with or without headaches, > 50 lifetime unilateral episodes	Unknown
Fuller et al. [29]	69m	No epilepsy; recurrent psychosis	Bilateral photopsia; tingling in lips and left arm	Well-formed, colourful images in motion involving “red and squirmy” piranhas on room floor, 100 burglars in front garden, hospital bed being swallowed up by floor or threatening words written on walls (engendering refusal to enter rooms); complaints that wife and brother-in-law were morphing, with lengthening arms and a single eye, lasting 7–28 days	None	None	Migraine with aura onset at age 16, episodes every 3–6 months, right-sided throbbing headaches	Before (30 min pain-free interval)
Hamed [30]	20m	No significant neurological or psychiatric history	Photopsia	Lilliputian hallucinations: Feeling like he was shrinking, and other persons were similar to size of his index finger; moving objects; voices of people varying in volume and distance; not associated with distress	None	None	No details provided	Unknown
Lindner et al. [31]	41m	No epilepsy	Scintillating scotoma in left visual field; geometric figures and wavy lines; homonymous hemianopia	Repetitive visual hallucinations every 5–7 min, lasting ~ 3 min, seen with closed eyes (except for phosphenes) in left visual field, such as moving streets and landscapes, emergency department nurse, Garfield cartoon image, phosphenes resembling American and German flags	Photophobia	Hypoesthesia/dysesthesia in left fingertips, forearm or perioral region	No details provided	Before
Lo et al. [32]	22m	Brainstem lesion; psychotic illness excluded	None	Heard people talking or shouting for up to an hour, individuals were having a conversation that clearly did not involve him	Photophobia	None	Migraine episodes twice a week, lasting up to 6 h, recurrent, pulsating right frontal headaches of moderate intensity, with nausea	Unknown
McAbee et al. [33]	16m	Circular mass in left posterior temporal region	Occasional blurred vision; left-sided numbness	Odour of “burnt wood”, lasting seconds to minutes	Photophobia; phonophobia	None	2-year migraine history, unilateral (primarily left temporal) severe pounding headache, lasting several hours, with nausea and vomiting	During; after
Miller et al. [34]^**ş**^	13–55 years old, 58% female (*n* = 12)	67% diagnosed with comorbid psychiatric disorder, most commonly MDD (1 suspected SCZ)	Photopsia and other visual symptoms (50%)	Single or multiple voices (58%), some of which were known; general noises (50%), like fridge humming, bells ringing, crickets, beeping or hissing; an example included evolution of hearing muffled hissing noises inside head, which clearly sounded like the word “death” following repetition; lasting < 1 h (67%)	Phonophobia (33%)	None	Mean migraine onset at age 16 (*n* = 8)	During (83% visual aura; 75% auditory hallucinations)
Partovi and Tolebeyan [35]	53f	None	None	Onset of olfactory symptoms for several years; associated with ~ 30–50% of episodes (persisted during COVID despite loss of olfactory abilities); odour of cigarette smoke from headache commencement for 3–7 days	Photophobia; phonophobia	None	Onset in early 20 s; 12 headache days a month, with pain (rated 4–9/10) on left side of neck and occipital area at times	Unknown
	48f	Anxiety and depression	None	Occasional odour of burning for ~ 30 min prior to headache onset (followed by visual symptoms); described as cigarette smoke or trash	Photophobia; phonophobia	None	Adolescent onset and progression from mid 30 s; described as pressure or sharp pain in occipital or frontal areas lasting up to 3 days; 15 headache days a month, usually occurring towards end of day	Unknown
Podoll and Robinson [36]	72f	No significant neurological or psychiatric history	None	Lilliputian hallucinations: Seeing increasing numbers of black beetles with faces running in a line across carpet and ceiling, occurring every 2–3 months, lasting 2–5 min, associated with insight and lack of distress, started at peak of severe headaches since early 30s	Photophobia; phonophobia	None	Migraine onset at age 10, with recurrent episodes diminishing from weekly to monthly with age; lasting 1–5 days, side-changing unilateral pulsating pain of moderate to severe intensity, exacerbated by stress	During
Roussos and Hirsch [37]	32f	No significant neurological history	Fortification spectra; perioral paresthesia extending from philtrum to chin	Odours of foods or cleaning products, of 5/10 intensity, occurring once per month, lasting ~ 1 min	Photophobia; phonophobia	Burning sensation in eyes and nose; generalised pruritus; tongue and neck pain; pallinosmia and smell distortions; metallic pallinugeusia	5-year history of migraine triggered by allium aromas; biparietal, crushing headaches, 10/10 pain intensity	Before (visual aura); during (burning sensation); after (paresthesia and other unusual sensory experiences)
Spranger et al. [38]	46m, 42m, 12m	Cerebellar atrophy (in two adults)	Distorted double vision; contralateral hemiparesis	Fully formed, colourful visual hallucinations in motion, alongside persecutory delusions, provoking intense anxiety; auditory hallucinations (in two adults)	None	Hemi-hypoesthesia	Unilateral severe throbbing headache, 2–3 episodes per year, lasting 3–14 days	After (within 24 h)
van der Feltz-Cornelis et al. [39]	40m	No epilepsy or narcolepsy; Psychotic disorder excluded	Photopsia; blurred vision	Voices suggesting to kill son, provoking anxiety and depressive symptoms, and sleep problems	None	None	Nocturnal migraine, several times per week, unilateral throbbing pain localised at temple	Before
Vreeburg et al. [40]	29m	Tonic–clonic seizures with LOC; no significant psychiatric history	None	Heard “plopping” sounds, followed by voices at times, involving several known individuals, of negative emotional valence, for example commands to jump in front of a train, occurring once a week, lasting several minutes	None	None	Occurs with or without headache, latter involves throbbing pain lasting up to two days	Before

*n* = number; m = male; f = female; TBI = traumatic brain injury; LOC = loss of consciousness; MDD = major depressive disorder, SCZ = schizophrenia

^a^Visual (e.g. scintillations, scotoma), sensory (e.g. pins-and-needles, numbness), speech and/or language, motor, brainstem (e.g. decreased consciousness, tinnitus, vertigo), retinal (e.g. monocular blindness)

^b^Prodromal symptoms may begin hours or days prior to migraine episode and can include sensitivity to light and/or sound, blurred vision or nausea, whereas postdromal symptoms may follow resolution of headache, persisting up to 49 h, and can include feeling tired or weary, concentration difficulties and neck stiffness. ^**ş**^Study combined case reports and participants from retrospective literature review, presented in a way that they could not be segregated from one another. Ear fullness is currently noted as established symptoms for other headache disorders, but not migraine with or without aura, in the ICD-3

Established aura symptoms were reported in all but five studies; visual symptoms usually involved some form of photopsia, scotoma and/or fortification spectra, whereas motor symptoms commonly related to paresis, numbness or tingling, typically in the perioral region or limbs, which could transpire uni- or bilaterally. The nature of complex hallucinatory experiences was diverse, and could include events in one or more of the major sensory modalities, namely, hearing voices or other sounds, smelling pleasant or unpleasant odours, accompanied by unusual tastes at times, or feeling touch sensations across the skin (as well as visual hallucinations), and more obscure experiences, such as Lilliputian hallucinations or perceived time travel. Documented phenomenological characteristics (e.g. frequency, level of conviction, functional impairment) were relatively scarce, though distress was explicitly noted in relation to auditory and (some) visual hallucinations. If present, prodromal or postdromal sensory symptoms commonly involved photophobia and/or phonophobia (*n* = 12). Other unusual sensory experiences, comprising osmophobia or hypoesthesia among others, were also described (*n* = 4).

In terms of associated migraine symptoms, age of onset or length of illness, headache frequency and duration, as well as pain quality, localisation and intensity all exhibited significant variation across participants. Hallucinatory (or unusual sensory) experiences mostly took place prior to headache onset, though some participants reported having these experiences during and/or after their headache (possibly depending on the nature of the sensory event).

### Multisensory hallucinatory and other unusual sensory experiences: group design studies

Details of established aura symptoms and the phenomenology of complex hallucinations and other unusual sensory experiences in migraine extracted from group design studies (*n* = 29) are presented in Table 2. Some unusual sensory experiences (such as ear fullness) outlined in Table 2 have been noted as established symptoms for other headache disorders, but are currently omitted from the diagnostic criteria for migraine [1]. Neurological and psychiatric comorbidities, associated migraine symptoms and relationships between migraine symptoms and aura/hallucinations have been placed in Table B of Supplementary materials, due to limited group design studies presenting these data.

**Table 2:** Multisensory hallucinatory and other unusual sensory experience in migraine (*n* = 29 empirical studies)

References	*N*, participant characteristics, age (*M* ± *SD* in yrs) and sex (% male)	Established aura symptoms (including visual; complex visual hallucinations presented in next column)	*Description of complex hallucinatory experiences (visual, auditory, olfactory, tactile, gustatory and other lesser-known modalities)	Unusual sensory experiences (prodromal or postdromal symptoms)	*Unusual sensory experiences (other)
Albanese et al. [41]	Mi+: *n* = 50, 36 ± 10, 30% HC: *n* = 58; 35 ± 8, 34%	*n* = 15 MA +; *n* = 35 MA− MA + had VA only	*N/A*	*N/A*	22% Mi + had ear fullness
Alstadhaug et al. [42]	*n* = 245; 55 ± 13, 62%; MA+: *n* = 95, 62%	98% of MA + had VA; ~ 8% of MA + had sensory auras; 14% of *n* = 245 had VA without headache; *n* = 10 had migrainous vertigo at least once	*N/A*	*N/A*	8% of *n* = 254 had UA + including distorted perception of time (*n* = 3), out-of-body sensation (*n* = 2), deja vu (*n* = 1), jamais vu (*n* = 1), microsomatognosia (*n* = 1)
Ashkenazi et al. [43]	*n* = 38; MA + *n* = 12; MA − *n* = 26, median = 26 (range 18–51); 21%	*N/A*	*N/A*	90% of AL + & 75% AL- had phonophobia; 97% AL + and 88% of AL- had photophobia; 53% of AL + rated phonophobia during Mi as moderately worse than phonophobia between Mi; 75% of AL- rated phonophobia during Mi minimally worse than phonophobia between Mi	35% of AL + and 25% of AL- had osmophobia during attacks
Baldacci et al. [17]	*n* = 100 Mi +; 39 ± 11; 33%	*N/A*	*N/A*	*N/A*	58% of *n* = 100 had osmophobia
Beh et al. [11]	*n* = 17 Mi +; Median = 49 (range = 17–63); 41%	47% had MA + (visual distortion): illusory splitting, teleopsia ("furniture seems far away, floor fells like a canyon"), underwater vision, xanthopsia ("yellow tint washing into visual field like a tide"), visual dolly zoom effect, frosted glass vision, enhanced stereoscopic vision ("seeing everything in extreme detail or extra 3D vision"), closed-eye visual hallucinations ("flashing images of unfamiliar people, trees, mountains, or buildings and phosphenes when eyes closed"). ~ 50% had visual distortion occurring during vestibular Mi, lasting for duration of Mi episodes (1–3 days); the distortion occurring between attacks usually lasted for mins to hours	100% Alice in Wonderland syndrome. Auditory distortion (“unable to hear own voice”) occurred during and lasted whole duration of Mi episodes (2–4 h). 41% had extrapersonal misperceptions: derealisation or out-of-body experience; most extrapersonal misperceptions occurred during and lasted whole duration of Mi. 29% had somesthetic distortion: aschematia “feeling as though [they] have no eyes but knows [they are] able to see”; partial macrosomatognosia – “brain becoming too big”; total body macrosomatognosia – “feels like [they are] growing so big [their] head would push through the roof”; microsomatognosia – “feels [they are] shrinking and becoming so small [they] would disappear behind the steering wheel”; most somesthetic symptoms occur during and lasted whole duration of Mi, except total body macrosomatognosia and microsomatognosia for *n* = 1 occurred between Mi episodes. 6% of Mi + experienced time distortion: "everything suddenly slowing down and moving very slowly even if [they were] driving at 80 mph on a highway"; occurred 1 day after Mi and lasted 5 min		
		MA + found these experiences more fascinating than worrisome			
Celebisoy et al. [44]	Vestibular M: *n* = 415, 42 ± 12 (17–74), 15%	10% of *N* reported hearing loss in audiometry; 41% of *N* reported tinnitus; 69% of *N* reported vertigo attacks lasting 60 min–24 h; 62% of *N* reported vertigo attacks associated with headache in > 50% of the attacks; median vertigo attack severity = 7^a^	*N/A*	42% of *N* reported photophobia or phonophobia	32% of *N* reported aural fullness; 20% of *N* reported osmophobia; 12% of *N* reported AL
Demarquay et al. [45]	*n* = 74 Mi + (*n* = 48 non-olfactory hypersensitive (OH-), *n* = 26 olfactory hypersensitive (OH +)), *n* = 30 HC; Mi + 42 ± 12; OH- 41 ± 13, OH + 44 ± 10, HC: 38 ± 12; Mi + 18%, OH- 15%, OH + 23%, HC 27%	23% of OH- had non-olfactory established aura; 15% of OH + had non-olfactory established aura	*N/A*	*N/A*	35% of Mi + had interictal olfactory hypersensitivity. OH + judged odours as less pleasant than OH- (*p* = 0.002) and controls (*p* = 0.006)
Dispenza et al. [19]	*n* = 30 Mi + with vertigo; 45 (range 18–64 yrs), 20%	*n* = 12 VA; all had vertigo; *n* = 10 had episodic tinnitus	*N/A*	*n* = 20 patients reported dizziness and unsteadiness. All had photophobia, *n* = 26 patients reported phonophobia	*n* = 20 had transitory ear fullness sensation, of which *n* = 9 had both symptoms after first pure tone audiometry testing
Gossrau et al. [46]	Mi: *n* = 113, 46 ± 14, 12%	44% of N reported Mi with aura	*N/A*	*N/A*	38%, 62% and 32% of *n* = 113 reported preictal, ictal and interictal hypersensitivity to odours respectively; common feared odours: perfumes, food odours and smoke (~ 35%, 22% and 12% of Mi + with osmophobia respectively)
Hansen et al. [47]	*n* = 267; MA +, 39 ± 11; 20%	Most common auras were dots or flashing lights (70%), wavy or jagged lines (47%), blind spots (42%) and tunnel vision (27%)	Visual hallucinations (*n* = 22). 19% of MA + experienced changes in smell	88% of Mi attacks were associated with photophobia and 73% with phonophobia	14% of MA + had changes in taste or touch
		Blurry vision (*n* = 22), photopsia (*n* = 14), halos (*n* = 7), obscuration (*n* = 4) and micropsia (*n* = 1). Most reported > one VA symptom (median = 2, range 1–5). 30% had numbness or pins and tingling			
Jürgens et al. [14]	*n* = 149 MA −, *n* = 70 MA +; *n* = 161 HC; MA− = 40; MA + = 39; HC = 42; MA− = 15%; MA + = 11%; HC = 20%	91% MA + reported VA symptoms. visual illusions: autokinesis, corona phenomenon, cinematographic vision, double vision, macropsia, micropsia, pelopsia, teleopsia, metamorphopsia, visual splitting, inverted vision, inversion of 2D/3D vision, dyschromatopsia and illusionary visual spread. 23% MA + had sensory aura symptoms	5% Mi + had complex visual hallucination. Mi + reported distorted perception: altered perception of body size, altered perception of body weight, altered perception of body position in space. Others: out-of-body experience, doppelganger phenomenon, synesthesia	*N/A*	*N/A*
Kandemir et al. [48]	MA+: *n* = 30, 34 ± 8, 13%; MA−: *n* = 30, 32 ± 8, 27%; HC: *n* = 30, 32 ± 7, 20%	*N/A*	*N/A*	*N/A*	77% MA + and 60% MA − reported osmophobia; perfume and cleaning products were the most common offensive odours during or between Mi attacks
Karli et al. [15]	*n* = 96 (*n* = 23 MA +, *n* = 33 MA −, *n* = 31 episodic tension-type headache (ETTH); *n* = 9 Aura without headache (A + H-)); MA + = 40 ± 11, MA− = 37 ± 10, ETTH = 35 ± 10, A + H− = 35 ± 10; MA + = 9%, MA− = 3%, ETTH = 23%, A + H− = 0%	44% of MA + had photopsia as VA; 33% of MA − also experienced photopsia before or during headache, but the symptom lasted < 5 min. 47% of MA + had fortification spectra. 39% of MA + had scotoma. 48% of MA + had paraesthesia, 12% MA − had paraesthesia within 1 h before or during headache, but the symptom lasted < 5 min	22% of MA + had olfactory hallucinations, 6% of MA − had olfactory hallucinations	Photophobia before headaches: 91% of MA +, 85% of MA −. Phonophobia before headaches: 87% of MA +, 91% MA −	13% of MA + had micropsia/macropsia; ↑ % of MA + felt cold before headache than MA −
Kayabaşoglu et al. [18]	*n* = 30 HC, *n* = 60 Mi +; HC range = 20–56 yrs, Mi + range = 20–54 yrs; HC = 40%; Mi + = 39%	*N/A*	*N/A*	*N/A*	Mi + detected odorant at ↓ odour concentration level than HC (threshold scores: 8 ± 2 Mi + with osmophobia; 8 ± 2 Mi + without osmophobia; 11 ± 1 HC, *p* < 0.001). Mi + with osmophobia had ↑difficulty discriminating odours. (discrimination scores: 6 + 1 Mi + with osmophobia; 9 + 1 Mi + without osmophobia; 12 ± 1 HC,* p* < 0.001). Mi + with osmophobia rated perfume (80%), smoke (70%), stale food (63%), fish (47%), coffee or spices (40%) and leather (40%) as a disturbing smell
Kelman [49]	*n* = 952 Mi +; 38 ± 12; 15%	92% of MA + had VA. VA was often the only aura symptom. 67% of MA + rated VA as very frequent. Out of total episodes of sensory aura (numbness and tingling), only 9% of sensory aura occurred in absence of VA. Those w/more frequent VAs were ↑likely to experience more frequent non-VA symptoms. 26% of MA + had frequent numbness or tingling sensation. *n* = 1 participant had hearing loss. *n* = 2 had tinnitus	*N/A*	When asked about “other aura symptoms before Mi”: *n* = 4 had photophobia	When asked about “other aura symptoms before Mi”: *n* = 1 patient reported vision jumping, “things go black. *n* = 1 had smell intolerance, strange smells. *n* = 1 had strange taste, metallic taste and acid taste. *n* = 1 had facial pain, flushing of face, chest tightness, body cold, neck tightness, head tightness, neck burning, stiff neck and shoulder burning
Leveque et al. [50]	MA−: *n* = 46, 29 ± 9, 22%; HC: *n* = 46, 27 ± 11, 28%	*N/A*	*N/A*	Mi + experienced ↑ sensory hypersensitivity than HC in the visual (*p* < 0.001) and auditory modalities (*p* = 0.002); visual hypersensitivity correlated with auditory in Mi + in interictal periods (*r* = 0.3, *p* = 0.0043) and ictal period (*r* = 0.432, *p* = 0.003)	Mi + experienced ↑ sensory hypersensitivity than HC in olfactory modality (*p* = 0.032); olfactory hypersensitivity correlated with auditory hypersensitivity (*r* = 0.315, *p* = 0.033) in ictal period
Mahmud and Sina [51]	MA+: *n* = 18; 30 (24–36), 17%; Patient with occipital epilepsy:* n* = 10, 22 (15–47), 20% age presented as median and IQR	Scotomas, white or golden dots, or light flashes lasting for minutes before the headache; median duration of the visual phenomenon = 460 s, IQR: 225–1800; vertigo (56% MA +) and tinnitus (6% MA +)		Photophobia (94% MA +), phonophobia (89% MA +)	Osmophobia (39% MA +); 2 MA + reported transient loss of consciousness as aura symptoms
Mainardi et al. [4]	Mi + with olfactory hallucinations: *n* = 11 (10MA −, 1MA +), 40 (25–36), 18%	*N/A*	Reported olfactory hallucinations included smell of burnt wood, vanilla, gas, jasmine, coffee, smoke, rotten meat, banana, bitter almonds, chemical, melon, metallic, sulphur. The smells were always the same for most participant. Average age of onset of olfactory hallucinations = 32 yrs (5–54); ~ 50% of *n* = 11 had sudden onset and stop of olfactory hallucinations; ~ 70% of *n* = 11 reported olfactory hallucinations lasted 3–10 min; all Mi + reported changing intensity of olfactory hallucinations during the attack (mild or extremely intense); ~ 70% of *n* = 11 considered the smell consistently unlikeable	Phonophobia and photophobia constantly accompanied the pain phase in all patients	50% of *n* = 11 reported osmophobia
Pekdemir and Tanik [52]	*n* = 145; Mi with osmophobia: *n* = 98, 36 ± 11, 14%; Mi with osmophobia: *n* = 47, 36 ± 9.26%	*N/A*	*N/A*	*N/A*	68% of Mi + reported osmophobia; median scores of ASC-12 (allodynia symptom checklist): Mi + with osmophobia = 5 (mild AL) and Mi + without osmophobia = 2 (no AL)
Petrusic et al. [53]	MA+: *n* = 40, 16 ± 2 (13–19), 50%	38% of MA + had VA only; 20% of MA + had visual and somatosensory auras; common VA included scintillating scotoma (67.5% of MA +), blurry vision (60%), tunnel vision (40%) and zigzag lines (25%); Feature of scintillating scotoma: onset time 21 ± 16 (25–60) mins in regard to the beginning of headache; duration 19 ± 17 (2–75) mins; 60% of MA + had somatosensory symptoms, including numbness in the left hand or both hands (53% of MA +), numbness in lips and/or face (30%), tongue (28%) and legs (15%)	*N/A*	*N/A*	23% MA + experienced deja vu phenomenon during the aura; 5% of MA + experienced neglecting hand syndrome (duration: 1 ± 18 (5–30) mins)
Price et al. [54]	Mi + with probable Mi: *n* = 117, 48 ± 14, 15%; HC: *n* = 827, 54 ± 17, 35%	*N/A*	*N/A*	Mi + have a ↑ level of sensory sensitivity in visual and auditory modalities than HC (*p* < 0.001)	Mi + have a ↑ level of sensory sensitivity in touch and taste modalities than HC (*p* < 0.001); sensory sensitivities in the different sensory modalities (visual, touch, taste and auditory) are correlated with each other (*p* < 0.01)
Saisu et al. [16]	*n* = 110, MA+: *n* = 31, 34 ± 10, 29%; MA−: *n* = 49, 35 ± 11, 23%; HC: *n* = 30, 35 ± 11.25%	*N/A*	No differences in gustatory sensation (hypogeusia or hypergeusia) found between Mi + and HC or between the MA + and MA −	66% Mi + reported photophobia and 74% Mi + reported phonophobia; ↑ MA + had photophobia and phonophobia than MA − (*p* < 0.05)	63% Mi + reported osmophobia: 45% MA + and 29% MA − found perfume offensive; 29% MA + and 5% MA − found cigarette smoke offensive; ~ 50% Mi + with osmophobia experienced osmophobia since the initial onset of Mi, before Mi attacks or within 30 min after attack onset; 71% Mi + with osmophobia had osmophobia stopped when the Mi ended; Mi + showed ↑ dislike towards odours of Japanese cypress, rose and perfume than HC (*p* < 0.05); MA + showed ↑ dislike towards clothes smelling of perspiration, rose scents and odours of cooking gas and curry than MA − (*p* < 0.05); 10% Mi + had dysgeusia
Shepherd and Patterson [5]	*n* = 151, MA+: *n* = 33, 39 ± 12, 27%; MA−: *n* = 44, 36 ± 11, 23%; HC: *n* = 74, 32 ± 8, 59%	3 MA + experienced tinnitus (buzzing in the ears); *n* = 9 MA + experienced dizziness or vertigo; 32 MA + experienced VA (included wavy lines, zigzags, squiggles, radiating lines, a sparkly big wheel, black and silver lines, flashes of dots, a scintillating scotoma, moving bright blue and black swirls like a lava lamp); most VA included motion, started small and centrally and then grow out to the periphery, superimposed on objects, obscuring them; 12 MA + experienced pins and needles or numbness, most commonly on one side of the face or on the nose, lips and mouth, less frequently on a hand or the fingertips, down one arm, leg or foot; Higher proportion of MA + experienced distorted or non-shared sensory experiences in visual modality (e.g. see shapes, lights, or colours when there is nothing there) than MA − and lastly than HC	↑ proportion of MA + experienced distorted or non-shared sensory experiences in auditory modality (e.g. hear noise and sounds that people near you do not; find sounds are distorted in a strange way), in olfactory modality (e.g. detect smells which do not seem to come from your surroundings), in tactile modality (feel someone is touching you but when you look nobody is there; burning sensation on your body), in gustatory modality (e.g. food or drink seems to have an unusual taste; unexplained tastes in your mouth) than MA − and lastly than HC	↑ proportion of MA + experienced visual and auditory sensitivity than MA − and lastly than HC	2 MA + experienced double vision; ↑ proportion of MA + experienced olfactory and tactile sensitivity (e.g. notice skin is more sensitive to touch, heat or cold than usual) than MA − and lastly HC
Shi et al. [55]	Vestibular Mi: *n* = 166, 43 ± 15 (6–76), 21%	52% of *N* had tinnitus; 21% of N had hearing loss- majority had mild and easily reversible low-frequency hearing loss; ↑ Mi + with hearing loss experience tinnitus versus those without (tinnitus = 80% of Mi + with hearing loss vs. 44% of Mi + without hearing loss); 14% of N had visual aura; most Mi + reported vestibular symptoms (incl. vertigo, dizziness and lightheadedness) monthly or yearly and lasted 5 min–72 h	*N/A*	32% of *N* had phonophobia; 24% of* N* had photophobia	8% of *N* had otalgia (majority one sided); 41% of *N* had aural fullness (majority one sided); ↑ Mi + with hearing loss experience aural fullness than those without (*p* < 0. 05) (aura fullness: 89% of Mi + with hearing loss vs. 29% of Mi + without hearing loss)
Silva et al. [12]	Sightless Mi+: *n* = 8, 40 ± 13 yrs (range 25–56), 33%	Short VA (< 5 min), with colour blue, silver or fire like and usually in round shapes,	Uncharacteristic noise	Photophobia reported by all Mi + whom the headache preceded the blindness in time (*n* = 6) disappeared after visual impairment	*N/A*
Sjaastad et al. [13]	*n* = 1838, mean = 43, 49%	10% of *n* = 1838 had visual disturbances: scintillating scotoma (62% of N with visual disturbances), obscuration (33%), photopsia (unformed flashes of light/star-shaped figures) (16%); photopsia coexisted with scintillating scotomas and/or obscuration occasionally; anopsia (dimness of vision) (5%), autokinesis (movement of stationary objects), tunnel vision and micropsia (< 5%); most of the rare visual disturbances (< 5% of N with visual disturbances) appeared before pain and appeared with other visual disturbances at the same time; 67% of those with visual experienced reported the experience lasted from > 15 to ≤ 35 min	One participant had red and hot ears bilaterally during Mi	*N/A*	*N/A*
		Paraesthesias usually occurred at face or upper extremity and occurred at multiple locations during Mi			
Teggi et al. [56]	Definite vestibular Mi+: *n* = 252, 46 ± 14 (range = 19–76), 15%	11% Mi + had tinnitus; 73% Mi + had internal vertigo – a false sensation of self-motion when no self-motion is occurring; ~ 50% Mi + with internal vertigo had spinning sensation; the age at the first vertigo attack = 38 ± 13 yrs (range 16–60 yrs.); vertigo usually lasted < 5 min (23% Mi +) or 6–60 min (22% Mi +)	*N/A*	Photophobia (44% Mi +), phonophobia (39% Mi +)	Fullness of the ear (9% Mi +)
Wang et al. [6]	MA−: *n* = 52, 24 ± 10; MA+: *n* = 28, 24 ± 10.00 (F), 19 ± 2 (M), 50%	MA + were ↑ likely to experience vision blurred and seeing black spot during Mi versus MA − (*p* < 0.01);	MA + were ↑ likely to experience the sensations that "head being hooped by a ribbon", "head being pressed by a big stone", "feeling like I was wearing hat" during Mi versus MA − (*p* = 0.01)	Blurred vision, light hypersensitivity (sensitivity to strong light when headache attacked), sound hypersensitivity	*N/A*
Zanchin et al. [57]	*n* = 1005, 37 ± 11, 23% MA−: *n* = 677, 38 ± 11, 20% MA+: *n* = 130, 36 ± 11, 27% Tension-type headache: *n* = 198, 36 ± 12, 31%	*N/A*	*N/A*	*N/A*	44% MA − and 39% MA + had osmophobia during Mi; For Mi + with osmophobia: 91% had osmophobia since first headache; 53% had osmophobia in 10/10 attacks; 73% had osmophobia during Mi; 98% had osmophobia stop with the pain phase; 43% had sensitivity towards two types of odours; most frequently reported offending odours: scents (e.g. feminine scents, deodorants, fresh flowers) (64% Mi +), food (e.g. coffee, fried foods, onion, etc.) (55% Mi +) and cigarette smoke (55% Mi +)

*n* = Number of participants; *M* ± *SD* = Mean ± standard deviation; HC = Healthy Controls; IQR = Interquartile Range; mins = Minutes; Mi = Migraine; Mi +  = People with migraine; MA +  = Migraine with aura; MA− = Migraine without aura; UA +  = Unusual aura; VA = visual aura; yrs = years

^a^Measured on a 0–10-point scale. Ear fullness is currently noted as established symptoms for other headache disorders, but not migraine with or without aura, in the ICD-3, and therefore was included as an “Unusual sensory experience (other)” for the purposes of the present study. Group specific information is reported if presented in this study

Of the group design studies, *n* = 17 reported established aura symptoms, including visual and sensory aura and vertigo. Descriptions of complex hallucinatory experiences varied, and commonly included auditory distortions (*n* = 3), tactile hallucinations (*n* = 3), Alice in Wonderland syndrome (*n* = 2), olfactory hallucinations (*n* = 2) or “changes” (*n* = 2). Auditory distortions included the inability to hear one’s own voice [11], or perceiving “uncharacteristic” or “distorted” noises [12]. Tactile hallucinations could involve feelings such as red, hot ears bilaterally [13], or one’s head being hooped by a ribbon, pressed by a stone, or feeling as though one was wearing a hat [6]. Coenesthetic hallucinations lasting the duration of the headache were also reported, where participants felt as though their brain was coming out of their head [11]. Reports of Alice in Wonderland syndrome included altered or distorted perception, involving one’s body size, weight or position in space [14]. Other distortions included extrapersonal misperceptions, such as out-of-body experiences and derealisation, typically lasting the duration of the headache [11]. Macrosomatognosia (e.g. feeling “like [they are] growing so big [their] head would push through the roof”) and microsomatognosia (e.g. feeling as though “[they are] shrinking and becoming so small [they] would disappear behind the steering wheel”) were reported, in addition to aschematia and time distortion [11]. Olfactory hallucinations could include the smell of foods/drinks (e.g. vanilla, melon, coffee, banana, bitter almonds), burning (e.g. burnt wood, smoke), chemicals/metals (e.g. gas, metallic, sulphur) or other negative smells (e.g. rotten meat). Individuals usually experienced the same olfactory hallucinations, with roughly half having sudden onset and remission. Most of these experiences lasted 3–10 min and were considered “unlikable” [4].

The overall frequency of migraine was found to be positively associated with the number of different types of unusual sensory symptoms experienced [14]. Compared to those without aura, individuals who experienced aura symptoms often reported a higher proportion of hallucinations or unusual sensory experiences, such as olfactory [15] or tactile [6] hallucinations, bodily temperature changes [15], “distorted” auditory, olfactory, gustatory or tactile events, or sensory hypersensitivities, such as osmophobia (*n* = 14; [5, 16]. Jürgens et al. [14] also reported that those with established aura had higher frequencies of autokinesis, corona phenomenon, cinematographic vision, metamorphopsia, dyschromatopsia, illusionary spread and synaesthesia. However, individuals without migraine aura still reported tactile [6] or olfactory hallucinations [15], as well as other unusual sensory experiences [5], including sensory hypersensitivities [15, 16], albeit at lower levels. People with migraine with osmophobia judged odours as less pleasant than those without olfactory hypersensitivity and reported significantly more painful and frequent headaches, as well as increased numbers of odour-induced migraines [17]. Some individuals with migraine also had more difficultly detecting and discriminating odours relative to healthy controls [18]. Aural fullness was outlined by limited studies (*n* = 3). The most common postdromal and prodromal symptoms were photophobia (*n* = 19) and phonophobia (*n* = 16). A large proportion of people with migraine with aura experienced headaches after the onset of the aura, of which most individuals experienced headache during the aura, or within 30 min after the cessation of the aura [19].

Overall, complex hallucinations and unusual sensory experiences in the context of migraine were diverse and numerous. Established visual aura were described as more fascinating than worrisome [11], but there were limited data regarding distress and functional impairment for the other sensory modalities. Distortions or hypersensitivities across diverse visual, auditory, olfactory, tactile and gustatory senses were evident, suggesting the presence of significant and varied sensory experiences, beyond typical established aura symptoms.

## Discussion

The current study represented a systematic review summarising the phenomenology of multisensory and other unusual perceptual experiences in migraine, outside the criteria of well-established aura, especially involving non-visual domains. Data were extracted from eligible case and group design studies, evaluated for quality based on requisite risk of bias assessments.

In response to our first research question, complex hallucinations were vividly described in more than half of all included studies (*n* = 29), involving auditory, olfactory, tactile, coenesthetic, gustatory modalities as well as other peripheral senses. Experiences within these dominant sensory modalities typically implicated hearing voices (or other sounds), smelling or tasting varied odours or flavours, or burning or touch sensations on one’s skin. Other unusual sensory experiences comprised Lilliputian hallucinations or Alice in Wonderland syndrome involving distortion of bodily perception, or less well-described perceptions implicating aural fullness, time distortions, out-of-body encounters or other anomalous events. However, existing accounts largely lacked systematic phenomenological information encompassing physical (e.g. frequency, duration), cognitive (e.g. controllability, insight) and emotional (e.g. distress, functional interference) experiential facets. This has rendered it difficult to draw consistent conclusions about the characteristics of these multisensory events. Other than isolated anecdotal accounts of distress associated with some forms of auditory (or visual) hallucinations, it was also hard to decipher which modalities or aspects were significantly associated with negative emotional outcomes or interfered with daily functioning in a way that may benefit from therapeutic intervention. One reason for this lack of attention could be that such aura symptoms are typically considered transient or reversible phenomena (though this does not preclude their potential for evoking distress, or persisting for a significant period). In fact, some migraine experiences could be mistaken for severe intoxication, psychiatric disorders or even a medical emergency. A possible exception was the study of olfactory hallucinations in migraine, which was methodically managed across a large-scale study providing rich phenomenological detail [4]. This study demonstrated that most participants did consider their olfactory experiences as unpleasant (although typical episodes were brief with sudden onset and comprising recurrent content).

In response to our second research question, other unusual sensory experiences (distinct from complex hallucinations) were noted in also more than half of all included studies (*n* = 29). Known prodromal or postdromal symptoms were reported, but there were also descriptions involving osmophobia (*n* = 15), as well as relatively rarer symptoms, which have yet to be classified as being nosologically associated with migraine. Focused investigations are therefore needed to determine how these symptoms should best be accounted for within a migraine disease framework. To address our exploratory research question, a cursory analysis of participant characteristics revealed no systematic patterns in terms of the age, sex or neurological and psychiatric comorbidities of affected individuals, and group design studies were also unable to establish consistent associations between hallucinatory experiences and specific migraine symptoms. Finally, our risk of bias assessments demonstrated that the limited conclusions drawn from our review were supported by the inclusion of studies largely of high quality (*n* = 40). Nevertheless, the presence of a number of lower quality studies (*n* = 11), coupled with limited data points pertaining to our variables of interest, signifies that future replication remains imperative.

### Clinical implications and recommendations

Despite the limited conclusive findings, there are still useful clinical lessons to be drawn from our review. First, multisensory complex hallucinations and/or unusual perceptual experiences, outside of the visual realm, affecting people with migraine are not uncommon. This can take place before, during and/or after the headache episode (if present), the temporal order of which could be specific to the individual or dependent on the type of experience. The unexpected onset and nature of these events can bring about surprise or even bewilderment in some affected persons. Furthermore, involvement of specific modalities (e.g. auditory) and/or content (e.g. unpleasant) is likely to trigger significant distress in some patients, although there could be other experiential facets (e.g. physical, cognitive or emotional) also directly related to negative emotional outcomes (but these remain unknown). By bringing attention to and directly asking about these experiences, it is hoped that improving clinician–patient awareness and communication may convey some benefit. In some cases, there may be scope for therapeutic intervention to manage patient distress. This can be in the form of basic psychoeducation, normalising to patients that such sensory perceptions can be part of the migraine episode, with no underlying sinister implications, and not to feel overly alarmed, where possible. Or in instances where the distress is overwhelming, appropriate referrals to mental health professionals for psychological treatment may be necessary.

Second, there are nosological implications worth further consideration. There is some incongruency in that complex visual hallucinations are currently captured within the diagnostic category of migraine with HIS [1], but complex hallucinations in other non-visual modalities are not accounted for anywhere in the current classification system. Visual hallucinations (and other visual symptoms) fall within the class of visual aura, but although there exists the label of sensory aura, this mostly pertains to sensations of numbness or pins-and-needles, neglecting complex non-visual hallucinations, even though these have clearly been reported by individuals with migraine. A similar case can be made for other unusual perceptual experiences, especially involving osmophobia, or even aural fullness. Akin to photophobia and phonophobia, these sensory hypersensitivities seemingly precede or ensue headache episodes as prodromal or postdromal symptoms, respectively, but have yet to be nosologically recognised within existing diagnostic criteria. Related to this, it is worth noting that the present study variously referred to aura, prodromal/postdromal and interictal symptoms, without fully separating these out at times. While it is important to delineate aura symptoms from those experienced during the prodrome/postdrome and interictal phases, the temporal course of these events is often complex and difficult to assess, with significant overlaps noted. Therefore, future research should seek to better delineate which symptoms fall under each (or a combination) of these phases for clearer nosological distinction (also see “Directions for future research” section).

The current findings may contribute to improved definitions of the neural effects of migraine and may aid in establishing migraine as more than a headache condition. Following these preliminary suggestions from the current review, added investigations to corroborate a robust empirical foundation supporting these recommendations, alongside open scientific debate by prevailing experts, would be constructive. Should an affirmative consensus be achieved, implementing these changes in the next classification revision would ensure nosological clarity and consistency moving forward. However, it remains important that any defined aura symptom should still meet ICHD-3 criteria.

### Study limitations

Our systematic review was subject to several limitations. Owing to the nature of our research questions, we had excluded studies focusing solely on sensory perceptual experiences already established within existing diagnostic criteria for migraine [1]. This largely comprised not only visual symptoms, whether in the form of complex hallucinations or other anomalous perceptions (e.g. photopsia, scotoma or fortification spectra), but could also include sensorimotor symptoms (e.g. paresis, numbness/tingling) as well as known prodromal indicators (e.g. photophobia or phonophobia). Such investigations did not contribute towards our consolidation of current knowledge to address existing gaps in the study of atypical non-visual sensory experiences in migraine (nb. the study of visual symptoms is evidently noteworthy, with *n* = 15 already identified in our search, suggesting it would necessitate a separate review). That being said, for completeness, we did extract sensory information pertaining to the visual realm, but only when this was presented alongside, in studies focusing on non-visual symptoms. Thus, any conclusions drawn from the current review need to be interpreted within this lens.

We also elected not to include an analysis of medication effects in our review, as this information had not been systematically collected across many of the included studies, meaning that any conclusions would likely have been biased in some way. However, given the possible impact of pharmacological drugs on unusual sensory experiences, this issue is deserving of detailed scrutiny under more opportune circumstances. Akin to this, a lack of consistent examination/associations between hallucinatory and migraine symptoms meant that we were unable to decipher meaningful patterns or arrive at more definitive conclusions in this regard (also see Table B in Supplementary materials). Phenomenological information, including frequency of modality-specific hallucinations as well as linking of timelines of unusual perceptual events relative to headache onset, especially key for mechanistic understandings, was only reported sporadically. We were thus unable to organise the extracted data in more meaningful ways, for instance, by ordering via magnitude of increasing frequency, according to prodromal versus postdromal occurrence, or clustering in line with similarity of experiences. On the other hand, our extensive search strategy, coupled with inclusion of a mix of study types, aimed at shedding light on this hitherto neglected area in migraine, is a key strength. Some of the most vivid descriptions of non-visual complex hallucinations tended to stem from case reports, whereas group design studies facilitated meaningful comparisons across subgroups with relatively robust participant numbers.

### Directions for future research

There are some fruitful avenues worth pursuing in terms of future research endeavours. Evidently, there needs to be more comprehensive investigations into all modes of unusual sensory experiences, including complex hallucinations, in the context of migraine. The prevailing emphasis on visual symptoms (although possibly more prevalent) at the expense of neglecting other sensory modalities denotes a bias that needs to be overcome via a more inclusive approach. This would comprise an examination of related phenomenological parameters (i.e. physical, cognitive and emotional) as well as patient characteristics and associated migraine symptoms. Given its less established nature in migraine studies, the validity of phenomenological inquiry of multisensory hallucinations may be buttressed by borrowing from appropriate existing measures in the psychosis field, or if necessary, creating new tools not premised on the dominance of visual sensory symptoms. Based on such studies, we may begin to elucidate which modalities/types of experiences are most prevalent and distressing, which subgroups of people with migraine are most likely to be affected (according to specific demographic or clinical features) and which key migraine symptoms can serve as prognostic indicators and/or therapeutic targets.

Related to this, tracking the temporal interaction of unusual sensory and migraine symptoms could be especially revealing. This could involve not just the timing of sensory and migraine symptoms relative to one another but also how such temporal ordering may evolve over time within individuals, potentially as a sign of migraine disease progression. From an etiological perspective, the involvement of multiple sensory processing systems before, during and/or after a migraine episode likely implicates the contribution of diverse neurobiological regions [20, 21]. Studying its overt manifestation in the form of unusual sensory perception may lead to improved mechanistic understandings that could culminate in therapeutic breakthroughs, especially if definitive neurobiological structures or causes can be isolated. Ensuing knowledge from these proposed lines of research will likely in turn bring about direct nosological and treatment benefits. Finally, taking into account the anomalous nature of such perceptual events, consideration should be given to investigating potential stigma in terms of reporting these phenomena as well as coping strategies. This could help raise awareness of these lesser-known migraine symptoms, possibly mistakenly associated with mental illness.

## Conclusions

The current systematic review aimed to provide a comprehensive documentation of multisensory complex hallucinations and other unusual perceptual experiences (beyond the visual domain) in the context of migraine. It was concluded that these experiences affect a certain proportion of people with migraine and vary widely in their presentation. This can range from typical everyday perceptions (e.g. smelling food scents) to extraordinary and vivid sensory events across one or more senses (e.g. hearing/seeing human interactions), or even border on the bizarre. It is likely that certain experiential modalities and/or facets are directly linked to patient distress, though added research is necessary to corroborate which phenomenological characteristics are most liable. This research contributes to a greater understanding of the neural effects of migraine and may help establish it as more than a headache condition. From a clinical perspective, increased clinician–patient awareness is vital to manage distress (where necessary), and scientifically, related nosological issues warrant further consideration. An inclusive management of these unusual sensory symptoms may be key to offering a holistic therapeutic approach in migraine. Given that precision medicine has been identified as a key next step in migraine diagnosis and management treatment [7], recognition of these understudied multisensory experiences is warranted.

### Supplementary Information

Below is the link to the electronic supplementary material.

Supplementary file 1 (DOCX 26 KB)

## Data Availability

For this systematic review data such as the search syntax, risk of bias assessment and further extracted data (not included in main tables) are presented in the Supplementary file 1.
