# Supplementary material for: Multisensory hallucinations and other unusual sensory experiences in the context of migraine: a systematic review
Source: J Neurol. 2024 Jan 18;271(4):1717–46. doi: 10.1007/s00415-023-12144-9 (PMC10972994; doi:10.1007/s00415-023-12144-9)
Supplement: Supplementary file 1 — (DOCX 26 KB) [file 415_2023_12144_MOESM1_ESM.docx]

**Supplementary Materials**

Table A

*Search syntax employed across PsycINFO APA and Web of Science*

| Database | Syntax |
| --- | --- |
| PsycINFO APA* | (title: migraine) And (abstract: “visual aura”) OR (abstract: “sensory aura”) OR (abstract: acoustic) OR (abstract: auditory) OR (abstract: sound*) OR (abstract: hear*) OR (abstract: olfactory) OR (abstract: smell*) OR (abstract: somatic) OR (abstract: feel*) OR (abstract: tactile) OR (abstract: touch*) OR (abstract: gustatory) OR (abstract: tast*) OR (abstract: hallucinat*) And (Any Field: Year: [2022 to 2023])  (title: migraine) And (abstract: “visual aura”) OR (abstract: “sensory aura”) OR (abstract: acoustic) OR (abstract: auditory) OR (abstract: sound*) OR (abstract: hear*) OR (abstract: olfactory) OR (abstract: smell*) OR (abstract: somatic) OR (abstract: feel*) OR (abstract: tactile) OR (abstract: touch*) OR (abstract: gustatory) OR (abstract: tast*) OR (abstract: hallucinat*)) And (Any Field: Year: [2021 to 2022])  (title: migraine) And ((abstract: “visual aura”) OR (abstract: “sensory aura”) OR (abstract: acoustic) OR (abstract: auditory) OR (abstract: sound*) OR (abstract: hear*) OR (abstract: olfactory) OR (abstract: smell*) OR (abstract: somatic) OR (abstract: feel*) OR (abstract: tactile) OR (abstract: touch*) OR (abstract: gustatory) OR (abstract: tast*) OR (abstract: hallucinat*)) And (Any Field: Year: [1980 to 2021]) |
| Web of Science* | TI=migraine AND AB=("visual aura" OR "sensory aura" OR acoustic OR auditory OR sound* OR hear* OR olfactory OR smell* OR somatic OR feel* OR tactile OR touch* OR gustatory OR tast* OR hallucinat*) AND Publication Date (2022-03-09 to 2023-02-01)  TI=migraine AND AB=("visual aura" OR "sensory aura" OR acoustic OR auditory OR sound* OR hear* OR olfactory OR smell* OR somatic OR feel* OR tactile OR touch* OR gustatory OR tast* OR hallucinat*) AND Publication Date (2021-01-01 to 2022-03-09)  TI=migraine AND AB=("visual aura" OR "sensory aura" OR acoustic OR auditory OR sound* OR hear* OR olfactory OR smell* OR somatic OR feel* OR tactile OR touch* OR gustatory OR tast* OR hallucinat*) AND Publication Date (1980-2021) |

*Note.* *Three-stage search.

Table B

*Neurological and psychological comorbidities, migraine symptoms, and associations between migraine symptoms and unusual sensory experiences or hallucinatory experiences (n=29 empirical studies)*

| **Author (year)** | **Neurological and/or psychiatric comorbidities** | **Associated Mi symptoms** | **Relationships between Mi symptoms and aura/hallucination** |
| --- | --- | --- | --- |
| Albanese et al. (2021) | *N/A* | 52% Mi+ had >4 Mi episodes monthly;  LOI 3-40 (17±12) yrs;  DPOAE (hearing) ↓ in Mi+ vs. HC *p*≤.01 | *N/A* |
| Alstadhaug et al. (2012) | *N/A* | *N/A* | ~50% of UA+ also had typical aura |
| Ashkenazi et al. (2010) | *N/A* | Median LOI=56 yrs (range=1-38 yrs); median frequency=3.5/month (range= 1-9/month); median Intensity^^^=8 (range=4-10) | *N/A* |
| Baldacci et al. (2015) | None | LOI=16±12 yrs; Frequency=7±6/month in previous 3 months. Intensity^^^=8±1 | Those with osmophobia had more painful Mi (*p*=.002) than those without osmophobia |
| Beh et al. (2018) | 43% with anxiety disorder, *N*=31% with depression, N=0% psychosis or neurological | Median age at onset=45 yrs (range=15-61 yrs). 24% reported Mi without aura prior to onset of vestibular Mi & Alice in Wonderland syndrome. |  |
| Celebisoy et al. (2022) | 36 patients have comorbid Meniere’s disease (9% of *N*) | Mean age at headache onset = 28±9 (range 10-42 years); Median Mi severity =8^^^; 76% of N reported duration of Mi episodes 4-24 hrs | Severe headache attacks (reported severity >6^^^ was correlated with the presence of allodynia (*p*=.006) |
| Demarquay et al. (2006) | *N*=0 with possible brain damage | Mi frequency: 5±3 days/month (OH-), 8±5 days/month (OH+)  LOI: 21±12 yrs (OH-), 21±13 yrs (OH+) | OH+ ↑ Mi frequency, OH+ ↑ number of odour-induced Mi, OH+ ↑ visual hypersensitivity (measured by numerical category scale) than OH-. |
| Dispenza et al. (2021) | *n=*26 reported anxiety. *N*=0 with previous diagnosis of Meniere's disease, vestibular schwannoma of VIII, head trauma, & diabetes, demyelinating disease, chronic renal failure. | Average LOI: 18 yrs before vertigo onset. Age at onset of aura was 21±12 yrs. 64% of Mi+ with headache had Mi aura with headache at every attack. 48% of MA+ with headache experienced 1-6 attacks within the last year. 55% of MA+ with headache experienced headache lasting 4-24 hours.  89% of MA+ with headache had moderate/ severe headaches. The age at onset of aura without headache (exclusively with aura) was 36±15, ↑than the age at onset of MA+ with headache (*p*<.0001) | 82% MA+ experienced headaches after the onset of the aura, among which most (96%) experienced headache during the aura or ≤ 30 mins after the cessation of the aura |
| Gossrau et al. (2022) | 3% of *N* reported anxiety disorder and 18% of N reported depression | *NA* | Mi+ with interictal hypersensitivity to odours on average experienced 8 years longer length of Mi disease vs. those without. (*p*=0.015) |
| Hansen et al. (2016) | *N*=0 with headache due to trauma | Age of onset: 18±9 yrs. LOI 21 yrs. Mi frequency: 4±2/month. Duration of Mi episodes: 22 hours (range 1-168 hours). Aura usually occurred during the wake hours, with peaks before noon & in early evenings. | N/A |
| Jürgens et al. (2014) | *N*=0 with pre-existing psychiatric or neurological comorbidity | Mi frequency (mean number/month for last 3 months): 8.5 (MA-); 6.5 (MA+). MA+ had ↑ types of unusual sensory experiences (mean=5) than MA- (mean=3), *p*<.001  ↑% of MA+ had autokinesis (*p*=.004), corona phenomenon (*p*<.001), cinematographic vision (*p*<.001), metamorphopsia (*p*=.015), visual splitting (*p*<.001), dyschromatopsia (*p*=.020), illusionary visual spread (*p*=.045) & synesthesia (*p*=.016) than MA-.  ↑% Mi+ had autokinesis (*p*<.001), corona phenomenon (*p*=.001), cinematographic vision (*p*<.001), metamorphopsia (*p*<.001), inversion of 2D/3D vision (*p*=.004), dyschromatopsia (*p*<.001), illusionary visual spread (*p*<.001), altered perception of body size & weight (*p*<.001), & synesthesia (*p*=.001) than HCs. | Number of headache days/month (Mi frequency) is +vely associate with # of unusual sensory symptoms (types) in all participants (*r*=.31; *p*<.001) & in Mi+ (*r*=.14; *p*=.034). Correlation was strongest in MA+ (*r*=.43; *p*<.001). |
| Kandemir et al. (2022) | *N*=0 with psychiatric or neurological comorbidities | *NA* | *NA* |
| Karli et al. (2005) | *N/A* | *N/A* | *N/A* |
| Kayabaşoglu et al. (2017) | *N*=0 with neuropsychiatric disease | *N/A* | *N/A* |
| Kelman (2004) | *N*=0 with complicated neurological problems were excluded | Non-visual aura rarely present without VA. In MA+, the mean % of aura occurrence with headache was 20% of headaches. Aura occurred exclusively before headache in 67%. Aura was followed by headache within 10 mins on average. | *N/A* |
| Leveque et al. (2020) | *N*=0 with psychiatric or neurological comorbidities | *N/A* | *N/A* |
| Mahmud and Sina (2022) | *N*=0 with occipital epilepsy | Median LOI = 30 months, IQR: 22-72; median duration of Mi episodes = 720 mins, IQR: 345-1440; headache was unilateral (44% MA+) or bilateral (50% MA+), & throbbing (72% MA+); median headache severity = 8, IQR: 7-8^^^ |  |
| Mainardi et al. (2017) | *N*=0 with neurological disease | Average Mi frequency= 3.9 attacks/month; average age at onset of Mi=17.81 yrs (4-31); 19% of Mi episodes were associated with olfactory hallucinations | Mi onset preceded olfactory hallucination onset by several yrs in most of Mi+, with an average interval between the two events =12 yrs. |
| Pekdemir and Tanik (2022) | *N*=0 with psychiatric or neurological comorbidities | *NA* | ↑Mi+ with osmophobia experienced nausea & allodynia vs. those without (*p*=.03, *p*<.001, respectively); measured via Allodynia Symptom Checklist |
| Petrusic et al. (2014) | *N*=0 with other neurological diseases, motor aura symptoms, & chronic Mi | 65% of MA+ reported onset of headache immediately after finish of aura. | *N/A* |
| Price et al. (2021) | *N/A* | *N/A* | *N/A* |
| Saisu et al. (2011) | *N/A* | Mi attack frequency: MA+ = 6 headache days/month; MA-=8 headache days/month | the degree of hedonic tone for nearly all odour types was ↑ for Mi+ with ≥ 6 Mi attacks/ month than for those with <6 attacks/month, with this tendency being especially prominent for the perfume, rose, cooking gas & Japanese cypress odours. |
| Shepherd and Patterson (2020) | *N*=0 with neurological or other condition that could affect visual acuity or day to day vision (e.g., photophobia, epilepsy, multiple sclerosis, diabetes, lupus & macular degeneration.) | Mi LOI: MA+ = 23± 15yrs (3-48); MA- = 19±14yrs (3-30); Mi frequency: MA+ = 44±48 episodes/yr (1-100); MA-= 32 ±31 episodes/yr (5-100); Mi+ & HC both reported anomalous perceptions but MA+ rated these experiences significantly ↑ distressing, & intrusive than HC. | tinnitus correlated with tactile sensitivity (experiencing one's skin to be ↑ sensitive to touch between Mi attacks) (*r*=.45, *p*=.01) |
| Shi et al. (2022) | *N*=0 with organic lesions or other vestibular disorder | Vestibular Mi onset of age= 36±14 (3-71) yrs; LOI 82±121 months | Mi+ with hearing loss tended to experience shorter duration of vestibular symptoms vs. those without (*p*<.05) |
| Silva et al. (2014) | *N*=0 with familial hemiplegic Mi, sporadic hemiplegic Mi, basilar-type Mi, retinal Mi, Mi complications, chronic Mi, migrainous infarction, seizure triggered by Mi, probable chronic Mi, Mi onset after the age of 50 yrs, & comorbidities potentially causing or manifesting as a Mi-like disorder | *N/A* | *N/A* |
| Sjaastad et al. (2006) | *N/A* | 78% of *N* with visual disturbances had visual phenomena followed by a pain-free interval & then headache | *N/A* |
| Teggi et al. (2018) | Excluded participants with mild low frequencies sensorineural hearing loss or microischemic lesions | Mi age of onset = 23±9 (range 11-40 yrs) | Mi+ w/ synchronous occurrence of headache & vertigo presented a ↓ age of onset of both disorders vs. others (20±2 yrs, *p*=.05); correlation between the age at onset of the first headache & the first vertigo= *r*=.37, 95% CI= .25-.46, *p*<.0001 |
| Wang et al. (2017) | *N*=0 with psychiatric or neurological disorders, head injury, alcohol or tobacco or substance abuse | *N/A* | *N/A* |
| Zanchin et al. (2007) | *N*= 0 with ≥ 2 primary headache | *N/A* | *N/A* |

*Note*. Group specific information is reported if presented in the study. *N*=Number of participants; *M*±*SD*=Mean±standard deviation; DPOAE=distortion product otoacoustic emissions; HC=Healthy Controls; IQR=Interquartile Range; LOI=Length of illness; mins=Minutes; Mi=migraine; Mi+=People with migraine; MA+=People with migraine with aura; MA-=People with migraine without aura; UA+=Unusual aura; VA=visual aura; AL= allodynia; yrs=years; ^^^ Measured on a 0–10-point scale.

Table C

*Risk of bias assessment for case report(s)/series using JBI-CAC (n=19 studies)*

| Author(year) | ***Case study or series*** | ***1. Was patient demographic characteristics clearly described?*** | ***2. Was patient history clearly described, with timeline?*** | ***3. Was patient migraine presentation clearly described?*** | ***4. Were diagnostic tests for migraine clearly described?*** | ***5. Were assessment methods for hallucinations adequate?*** | ***6. Was information descriptive enough for phenomenology?*** | ***Total scoring (out of a maximum of 12)*** |
| --- | --- | --- | --- | --- | --- | --- | --- | --- |
| Alstadhaug and Benjaminsen (2010)  Barros et al. (2012)  Bhatia et al. (2008)  Burstein et al. (2000)  Chen et al. (2012)  Donat and Donat (2008)  Fisher (1986)  Fuller et al. (1993)  Hamed (2010)  Lindner et al. (1996)  Lo et al. (2011)  McAbee et al. (2000)  Miller et al. (2015)  Partovi and Tolebeyan (2022)  Podoll and Robinson (2001)  Roussos and Hirsch (2014)  Spranger et al. (1999)  van der Feltz-Cornelis et al. (2012)  Vreeburg et al. (2016) | Study  Series  Study  Study  Study  Study  Study  Study  Study  Study  Study  Study  Series  Series  Study  Study  Series  Study  Study | 2  2  2  2  2  2  2  2  2  2  2  2  2  2  2  2  2  2  2 | 2  0  2  2  2  0  2  2  2  2  0  0  2  2  2  2  0  2  2 | 2  2  2  2  2  2  2  2  2  2  2  2  2  2  2  2  2  2  2 | 2  2  2  2  2  2  1  2  2  2  2  2  2  2  2  2  2  2  2 | 1  1  1  1  1  1  1  1  1  1  1  1  1  1  1  1  1  1  1 | 2  2  2  2  2  2  2  2  2  2  2  2  2  2  2  2  2  2  2 | 11  9  11  11  11  9  10  11  11  11  9  9  11  11  11  11  9  11  11 |

*Note.* JBI-CAC=JBI Critical Appraisals Checklist for Case Studies. For all six items, 2=Yes, 1=Maybe or unclear, 0=No.

Table D

*Risk of bias assessment for group design studies using NOQAS (n=29 studies)*

| **Author( year)** | **Is case definition (i.e. diagnostic criteria) adequate? *(2=Yes, migraine diagnosis with independent validation; 1=Yes, migraine diagnosis based on record linkage or self-report; 0=No description of migraine diagnosis)*** | **Representative-ness of cases *(1=Truly or somewhat representative of average in target migraine population; 0=Potential for selection bias or not stated)*** | **Selection of controls *(2=Yes, control group selected; 1=not applicable, or control group not relevant to research questions; 0=No control group or no description)*** | **Definition of controls *(2=Both clinical and non-clinical controls; 1=Either clinical or non-clinical controls; 0=No control group, or no description)*** | **Comparability of cases and controls on basis of design or analysis *(1=Matches cases and controls in design or analysis; 0=Does not match groups or no description)*** | **Validity of hallucinations assessment *(2=Gold standard or validated measure; 1=Clinician rating or unvalidated measure; 0=Other non-standardised measure or no description)*** | **Validity of migraine symptom assessment *(2=Gold standard or validated measure; 1=Clinician rating or unvalidated measure; 0=Other non-standardised measure or no description)*** | **Total scoring (out of a maximum of 12)** |
| --- | --- | --- | --- | --- | --- | --- | --- | --- |
| Albanese et al. (2021) | 2 | 1 | 2 | 1 | 1 | 1 | 1 | 9 |
| Alstadhaug et al. (2012) | 2 | 1 | 1 | 0 | 0 | 1 | 1 | 6 |
| Ashkenazi et al. (2010) | 2 | 1 | 2 | 1 | 1 | 1 | 1 | 9 |
| Baldacci et al. (2015) | 2 | 1 | 2 | 1 | 1 | 1 | 1 | 9 |
| Beh et al. (2018) | 2 | 1 | 1 | 0 | 0 | 1 | 1 | 6 |
| Celebisoy et al. (2022) | 2 | 1 | 2 | 1 | 1 | 1 | 1 | 9 |
| Demarquay et al. (2006) | 2 | 1 | 2 | 2 | 1 | 2 | 1 | 11 |
| Dispenza et al. (2021) | 2 | 1 | 1 | 0 | 0 | 1 | 1 | 6 |
| Gossrau et al. (2022) | 2 | 1 | 2 | 1 | 1 | 1 | 1 | 9 |
| Hansen et al. (2016) | 2 | 1 | 1 | 0 | 0 | 1 | 1 | 6 |
| Jürgens et al. (2014) | 2 | 1 | 2 | 2 | 1 | 1 | 1 | 10 |
| Kandemir et al. (2022) | 2 | 1 | 2 | 2 | 1 | 1 | 1 | 10 |
| Karli et al. (2005) | 2 | 1 | 2 | 1 | 1 | 1 | 1 | 9 |
| Kayabaşoglu et al. (2017) | 2 | 1 | 2 | 2 | 1 | 2 | 1 | 11 |
| Kelman (2004) | 2 | 1 | 1 | 0 | 0 | 1 | 1 | 6 |
| Leveque et al. (2020) | 2 | 0 | 2 | 1 | 1 | 1 | 2 | 9 |
| Mahmud and Sina (2022) | 2 | 1 | 2 | 1 | 1 | 1 | 1 | 9 |
| Mainardi et al. (2017) | 2 | 1 | 1 | 0 | 0 | 1 | 1 | 6 |
| Pekdemir and Tanik (2022) | 2 | 1 | 2 | 1 | 1 | 1 | 1 | 9 |
| Petrusic et al. (2014) | 2 | 1 | 2 | 1 | 0 | 1 | 1 | 8 |
| Price et al. (2021) | 1 | 1 | 2 | 1 | 0 | 2 | 2 | 9 |
| Saisu et al. (2011) | 2 | 1 | 2 | 2 | 1 | 2 | 1 | 11 |
| Shepherd and Patterson (2020) | 2 | 1 | 2 | 2 | 1 | 2 | 1 | 11 |
| Shi et al. (2022) | 2 | 1 | 2 | 1 | 1 | 1 | 1 | 9 |
| Silva et al. (2014) | 2 | 1 | 1 | 0 | 0 | 1 | 1 | 6 |
| Sjaastad et al. (2006) | 2 | 1 | 1 | 0 | 0 | 1 | 1 | 6 |
| Teggi et al. (2018) | 2 | 1 | 2 | 1 | 0 | 1 | 1 | 8 |
| Wang et al. (2017) | 2 | 0 | 2 | 1 | 1 | 2 | 2 | 10 |
| Zanchin et al. (2007) | 2 | 1 | 2 | 1 | 1 | 1 | 1 | 9 |

*Note.* NOQAS=Newcastle-Ottawa Quality Assessment Scale. For items in the table, 2 = favourable condition, 1= partially favourable condition, 0=unfavourable condition.
